# The Experience of an Accelerated COVID-19 Immunization Campaign in Oman: A Review Within the WHO Health System Building Blocks Framework

**DOI:** 10.3390/vaccines13101002

**Published:** 2025-09-25

**Authors:** Thamra Al Ghafri, Lamya Al Balushi, Zainab Al Balushi, Lamees Al Kiyumi, Asma Bait Ishaq, Jannat Al Harthi

**Affiliations:** 1 Directorate General of Health Services, Ministry of Health, Sultan Qaboos University, Muscat, Oman; 2 Directorate of Disease Surveillance and Control, Ministry of Health, Muscat, Oman; lamya2282@gmail.com (L.A.B.); drznoorf@gmail.com (Z.A.B.); 3 Directorate of Nursing, Ministry of Health, Muscat, Oman; lamees.alkiyumi@hotmail.com; 4 Directorate of Pharmacy and Medical Supplies, Ministry of Health, Muscat, Oman; baitishaqasma@gmail.com; 5 The College of Medicine and Health Sciences, National University of Science and Technology, Muscat, Oman; jannatalharthi@gmail.com

**Keywords:** COVID-19 vaccination, mass vaccination, WHO health system building blocks, vaccine hesitancy, primary healthcare, Oman

## Abstract

Oman launched its COVID-19 vaccination campaign in December 2020, navigating significant public health challenges with resilience and adaptability. The country faced global vaccine shortages, community hesitancy to receive the vaccine, and diverse sociocultural and infrastructural obstacles. Despite these issues, Oman scaled up its COVID-19 vaccination efforts, administering over 7 million doses, covering approximately 71% of the population by mid-2022. The campaign, which operated through more than 44 vaccination centers nationwide, exemplified rapid vaccine implementation, strategic prioritization, and a coordinated pandemic response. This study examined the field experience of Oman’s accelerated COVID-19 mass vaccination campaign through the World Health Organization’s Health System Building Blocks framework. The key domains addressed included (1) multi-sectoral collaboration underpinned by strong governance structures; (2) the role of primary healthcare facilities as essential first responders during health crises, and safe handling of vaccination procedures; (3) transparency and active community engagement, particularly through local leaders and social media, to address vaccine hesitancy; (4) the integration of digital health information systems to ensure unified and efficient data management; (5) building a resilient healthcare workforce by enhancing vaccination capacity and mental health support; and (6) the importance of financial alternatives. Additionally, the critical role of global and regional partnerships in vaccine production and distribution was highlighted. Drawing on Oman’s experience, this descriptive review offers context-specific lessons for enhancing health system preparedness and guiding effective responses to public health emergencies.

## 1. Introduction

In March 2020, the World Health Organization (WHO) officially declared COVID-19 a global pandemic, labeling it as a public health emergency with anticipated severe social and economic consequences [1]. The rapid spread of the virus underscored the urgent need for coordinated responses, ranging from preventive measures to large-scale healthcare, including COVID-19 vaccinations [2,3]. The effectiveness of vaccinations in alleviating the consequences of COVID-19, in both individuals and communities, has been reported in numerus studies, even with a one-dose strategy [1,2].

In 2016, Oman scored 99% out of a total score of 127 across all criteria in the global effective vaccine management (EVM) assessments conducted by the WHO and UNICEF in 90 countries [3]. This established a strong foundation for national vaccination campaigns that aimed to ensure quick delivery of high-quality vaccines during the COVID-19 pandemic. Additionally, Oman has ongoing collaborations with major international bodies that influence vaccine access and immunization policy, maintaining coordination and consultative negotiation channels with Gavi, the Vaccine Alliance (a global public–private partnership that helps improve access to vaccines, especially in low- and middle-income countries), and other vaccine manufacturers to ensure vaccine availability during emergencies [4].

**Figure 1:**
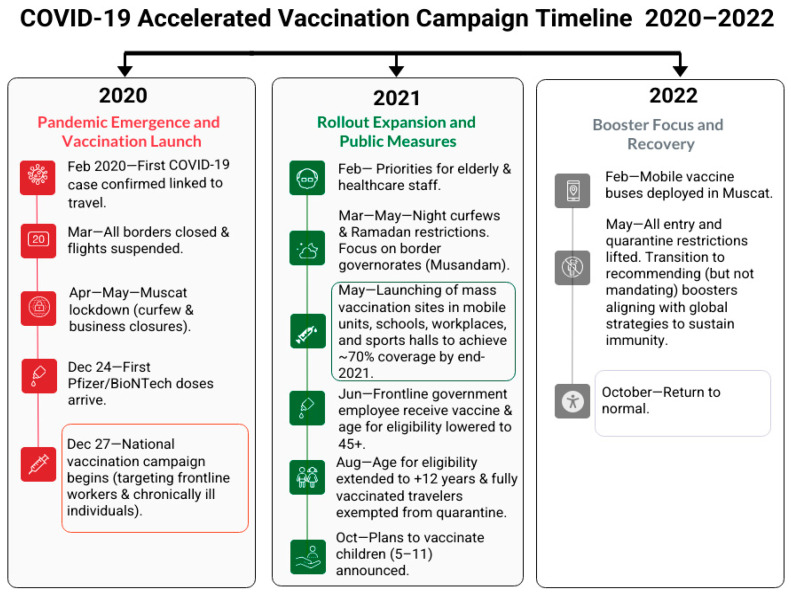
Accelerated COVID-19 vaccination campaign timeline and activities from 2020 to 2022.

Oman launched its COVID-19 vaccination campaign in December 2020, implementing a structured and phased approach to immunizing its population (Figure 1) [5]. Initially, the vaccination effort prioritized high-risk groups due to limited vaccine supplies and high global demand. This priority framework targeted vulnerable populations, including frontline healthcare workers and individuals with chronic health conditions, to mitigate the immediate impact of the pandemic. Following this, Oman established a strategic objective to vaccinate 70% of its total population, which would occur in two phases: the first phase aimed to cover 30% of the population starting in late 2020, and the second phase planned to reach the remaining 40% from July to October 2021 [6].

To facilitate this extensive rollout, Oman established mass vaccination centers throughout its governorates and collaborated closely with the private sector, enhancing accessibility and distribution efficiency. However, despite these efforts, the campaign faced significant challenges. These included limited vaccine production capacity and issues with timely procurement, exacerbated by difficulties obtaining pre-booked vaccines through the global COVID-19 Vaccines Global Access (COVAX) initiative, the campaign necessitated the direct purchase of vaccines from vaccine manufacturers at much higher prices than those booked through COVAX [6,7,8]. This urgent action led to the reallocation of government budgets and prompted immediate cost–benefit analyses to prioritize funding plans and balance timely access with financial sustainability.

A case study from Musandam, a border region in Oman, highlighted the success of rapid vaccine deployment in prioritizing first-dose administration in a vulnerable community [9]. Within a month, vaccine coverage in this region surged from 20% to 58%, correlating with a marked reduction in community transmission and a 75% decrease in COVID-19-related hospitalizations. This targeted, expedited approach provided critical evidence for the effectiveness of prioritizing high-risk border areas in curbing the pandemic’s health impacts [8,9].

Vaccine hesitancy was addressed using multiple strategies aimed at building public trust and encouraging vaccine uptake. Key government and community figures, including the Minister of Health, were prioritized to receive the vaccine early, serving as advocates and role models to promote public confidence. Information campaigns disseminated data on the vaccine’s effectiveness both globally and within the country, utilizing multiple media platforms to reach diverse audiences. Success stories from other nations that had managed to control the pandemic through vaccination were publicized to reinforce positive perceptions of the vaccine. Initially, vaccination updates were shared daily, which later shifted to a weekly schedule, paired with reports that highlighted improvements in the national epidemiological situation. As an incentive, fully vaccinated individuals without symptoms were exempted from testing and quarantine requirements. Additionally, implementing vaccination mandates for entry into public events, educational institutions, and workplaces proved influential in motivating the public to get vaccinated. These combined strategies effectively contributed to overcoming reluctance and boosting vaccine acceptance.

According to Reuters research, Oman administered at least 7,068,002 doses of COVID-19 vaccines during the pandemic’s peak [10,11]. Assuming two doses per person, this amount was sufficient to vaccinate about 71% of the country’s population by 15 July 2022, from a total population of about 4.98 million, as confirmed by Gulf Cooperation Council (GCC) reports [12]. This rapid vaccination rollout positively correlated with low COVID-19 mortality rates in vaccinated populations (<1%), even during surges in the Omicron variant [12,13,14,15,16,17,18,19,20,21].

## 2. The Objective of the Review

This descriptive review employs the WHO Health System Building Blocks framework, which includes six core components, to describe Oman’s accelerated COVID-19 mass vaccination campaign from February 2020 to October 2022, highlighting its strengths and challenges, and the key lessons learned (Table 1). The insights gained aim to enhance public health emergency preparedness and inform national response policies. All authors participated directly in the vaccination campaign at various managerial levels, including offering national expertise, providing firsthand experience that underpins this manuscript. Similarly, to existing reviews [22,23], this paper aims to document Oman’s unique experience of the COVID-19 pandemic and contribute to the global knowledge base on mass vaccination during pandemics. Our team combined expertise in policy planning, implementation, data management, and community engagement to comprehensively analyze the campaign through the WHO framework, with ongoing internal discussions ensuring accuracy and a holistic perspective.

## 3. The WHO Health System Building Blocks Framework

The WHO Health System Building Blocks framework offers a systematic approach to analyzing and strengthening health systems. It identifies the following six core components that interact improve health outcomes, equity, responsiveness, financial risk protection, and efficiency [24]: leadership and governance, which includes policy guidance, system supervision, accountability, and strategic alignment; service delivery, which emphasizes the provision of safe, effective, and quality health services to those in need; health workforce, which emphasizes the availability, competence, and equitable distribution of trained personnel; health information systems, which involves the generation, examination, analysis, distribution, and use of reliable data for decision-making; access to essential medicines, vaccines, and technologies, which ensures that critical health products are available, affordable, and of assured quality; and health system financing, which addresses the mobilization and allocation of resources to provide services without causing financial strain. The framework underscores that these components are interconnected, and weaknesses in one area can undermine the overall system’s performance in various contexts. Applying the building blocks in policy and program evaluation helps identify gaps, prioritize interventions, and promote resilience, particularly during public health emergencies such as the COVID-19 pandemic [25,26]. Its adaptability renders it a valuable tool for guiding national and international health initiatives.

## 4. Assessing the Accelerated COVID-19 Vaccination Campaign in Oman in Relation to the WHO Health System Building Blocks

### 4.1. Leadership and Governance

A Supreme Committee including relevant stakeholders from different sectors was formed in March 2020 to deal with progression of the COVID-19 pandemic, with multi-stakeholder arrangements made to accelerate the rollout of vaccines and sustain their coverage throughout 2021–2022. Priorities were focused on harmonizing responsiveness to the pandemic while minimizing its impacts on health, society, and the economy. Strong political will, governed by the Ministry of Health (MoH), led a unified strategy supported by inter-ministerial collaboration to empower national and regional teams to act promptly in accelerating vaccination efforts.

The MoH led a unified strategy, supported by inter-ministerial collaboration, to enable national and regional teams to work towards accelerating their vaccination campaigns. With the launch of COVID-19 vaccinations on 27 December 2020, the prioritization of healthcare workers (HCWs), high-risk groups, and border regions (Musandam governorate, a high-risk border region) was a strategy employed to improve the protection of the healthcare workforce [3]. Decentralized leadership enabled enhanced assessments of HCWs’ exposure risk, deployment of outreach teams, facilitation of public–private partnerships (PPPs), and dissemination of information to the public, while weekly press conferences led by the Minister of Health effectively countered misinformation, reduced vaccine hesitancy, and encouraged vaccine acceptance. Public knowledge of the vaccination of government and community leaders built confidence in vaccine safety and efficacy, and transparent monitoring and reporting of vaccine side effects were actively promoted [13,27].

The Supreme Committee members addressed vaccine hesitancy by responding to public questions collected via the MoH Twitter account prior to press conferences, while social media platforms and community influencers played vital roles in disseminating accurate information and countering misinformation. Incentives such as waiving quarantine for vaccinated individuals and mandating vaccination for access to public spaces boosted vaccine uptake, and a 24/7 MoH call center and telemedicine service addressed public inquiries on vaccinations throughout the pandemic [28].

In comparison to other GCC countries, Oman’s COVID-19 vaccination response exhibited a higher government Stringency Index score of 63.1 and more rapid policy initiation, reflecting strong and timely leadership [12,29]. The Stringency Index measures the strictness of government policy on containment and closure on a scale of 0 to 100, while the Initial Response Index quantifies the promptness and intensity of early COVID-19 actions [30]. Oman scored 45.7 in the latter, the highest among the GCC states. Regarding vaccination efforts, Oman’s Vaccine Index, an aggregate measure reflecting vaccination coverage and rollout efficiency, ranked fourth on average within the GCC, yet reached a sustained peak of 100 from late December 2021, indicating full coverage and efficient distribution [12,16,21].

#### 4.1.1. Roles of Other Core Government Sectors

Under health sector governance, other sectors played vital roles in maximizing vaccine coverage. The education sector provided school sports halls and university spaces to be employed as vaccination centers and facilitated rapid training of healthcare personnel, bolstering vaccination capacity. Logistics and transportation efforts included the establishment of drive-through vaccination facilities by the Oman Automobile Association and the assurance of timely vaccine importation and cold-chain integrity by aviation authorities ensuring.

Security agencies such as the Royal Oman Police enforced movement restrictions and ensured safety at vaccination sites, while the tourism and hospitality sector contributed venues for quarantine and vaccination, expanding vaccination capacity. In addition, media and risk-communication strategies, coordinated with government agencies, disseminated accurate information, countered misinformation, and promoted vaccine acceptance [4,6,7,8].

#### 4.1.2. Integrating Public–Private Partnership

During COVID-19 surges, private health providers supported outreach vaccination and assigned their staff to public centers [31]. For example, the Badr Al Samaa Group operated large vaccination centers administering thousands of Pfizer doses to employees from over 450 companies [32]. Moreover, employer-paid vaccinations were administered at private hospitals, facilitated by MoH grants [33,34]. Additionally, corporate social responsibility funding filled vaccine delivery gaps, with contributions from Occidental Oman (OMR 1 million, ~USD 2.5 million) [35], Oman LNG (OMR 6 million, ~USD 15.6 million) [36], and the Oman Chamber of Commerce and Industry (OMR 1 million, ~USD 2.6 million) [37], which also encouraged the prioritization of staff vaccination. In addition, commercial banks and employers organized on-site or clinic-based vaccinations, often in coordination with private hospitals [33].

#### 4.1.3. Role of the Community and Volunteers

Community and volunteer engagement in public health efforts represents a long-standing tradition in Oman [38]. Since 1991, the MoH has promoted community participation models emphasizing local collaboration in public health initiatives. Prior experience from outbreaks, like H1N1 in 2009, and vaccination campaigns against diseases such as measles have strengthened Oman’s pandemic response [7,39]. From early 2020 to mid-2022, communities played pivotal roles in adopting preventive measures, supporting healthcare adaptation, and disseminating accurate information, helping reduce global vaccine hesitancy [8,20,21,40]. Adherence to non-pharmaceutical interventions like social distancing, mask wearing, and hand hygiene substantially limited disease spread, supported by government regulations and initiatives such as “healthy cities and villages” [8,41,42].

Examples include the Al Buraimi healthy city “We are all responsible” campaign, which raised awareness during religious events, and Nizwa’s healthy city initiative of fostering civil society–academic collaboration to produce and distribute reliable educational materials [6,42]. Public messaging addressed preventive measures and healthy lifestyles, with information disseminated via billboards and social media, and community participation was encouraged through seminars and virtual meetings, promoting dialog and the sharing of coping strategies [7,43].

Local Wilayat health committees facilitated multisectoral collaboration by identifying challenges and proposing community-based solutions. Moreover, civil society organizations and volunteers mobilized resources, distributed medical equipment, and supported vulnerable groups at home, and the geriatric civil society’s provision of home oxygen concentrators reduced hospital burden, exemplifying effective community involvement in pandemic management [42].

### 4.2. Service Delivery and Healthcare Responsiveness

Multi-sectoral vaccination sites such as drive-through facilities, mobile units, and home services targeting elderly and vulnerable individuals were employed to enhance access to vaccines, while volunteers and NGOs managed mass gatherings and facilitated crowd control [3]. Moreover, efficient triaging for high-risk cases was established, and hospitals were restructured to comply with infection control protocols [7].

Primary healthcare (PHC) services played a central role in implementing clinical and safety measures across vaccination sites, with patients following structured screening, physical distancing, and mask use protocols. A smart queuing system called clients to booths, where trained nurses administered vaccines. Post-vaccination observation lasted about ten minutes, after which clients received certificates of vaccination and booked follow-up appointments. The entire process took 20–30 min on average [44]. Despite PHC’s key role in screening, case detection, psychosocial support, and demand mitigation, documentation of its role in the literature remains limited [45].

#### 4.2.1. Evidence for COVID-19 Vaccination

Vaccinated individuals received electronic COVID-19 vaccination certificates via the “Tarassud+” system. These included personal and vaccine details with digital verification to prevent fraud, accessible for domestic use and international travel. These certificates enabled authorities to securely verify vaccination status through encrypted codes or QR codes linked to the national database, ensuring transparency while prioritizing data privacy and accuracy [3].

#### 4.2.2. Reporting of Reactions to COVID-19 Vaccination

Following vaccination, individuals were encouraged to report any adverse reactions, ranging from common mild side effects to rare serious events. The MoH implemented a structured surveillance system supported by digital platforms, primarily the “Tarassud+” system, to collect and monitor these reports in real time.

Studies conducted in Oman, namely at Sultan Qaboos University Hospital, revealed that reported adverse drug reactions (ADRs) were mostly mild, including symptoms like fever, chills, localized pain, and swelling at the injection site [2,13,14,27,41]. The data showed a higher prevalence of reactions reported among females compared to males, consistent with global trends. Serious adverse events were rare, but the system was designed to ensure immediate follow-up and referral for any patients experiencing severe or unexpected reactions, strengthening clinical safety oversight [13,46]. Cutaneous reactions were also monitored carefully within specific governorates, with cases documented through prospective observational studies that aided in understanding vaccine safety at a population level. Notably, the government linked the reporting of adverse events with extensive public education campaigns and weekly Supreme Committee reports to counter misinformation and address vaccine hesitancy by sharing safety data [13,46].

Oman’s pharmacovigilance aligned with international standards, integrating data on adverse events with digital certificates and thus supporting proactive, data-driven safety monitoring, which reinforced public trust during the campaign [8,47,48].

#### 4.2.3. Reaching out to Non-Arabic Speakers

To ensure equitable access, the “Tarassud+” app was available in Arabic, English, Urdu, Hindi, and Bengali, catering to expatriate populations [41]. This multilingual digital platform provided timely updates on cases, testing, vaccination, and guidelines. Community engagement with expatriate leaders fostered culturally sensitive communication and trust, dispelling misinformation and encouraging vaccination. Public health messaging was disseminated via social media, radio, and television in the relevant languages, while vaccination centers provided multilingual staff and interpreters to assist non-Arabic speakers, ensuring service clarity. Moreover, coordination with foreign embassies supported multilingual efforts and access to healthcare, with regular sharing of epidemiological data strengthening cooperation [7,8,38].

### 4.3. The Health Workforce

Rapid training programs, especially using online and virtual platforms, were put in place regarding vaccine administration and safety protocols, with tasks shifting to non-physicians to expand vaccination capacity. To address staff burnout caused by intense workloads, prolonged working hours, and psychological strain during the campaign, the strategy included timely workforce planning and the integration of mental health support services. Public–private partnership (PPP) donations and volunteer involvement, including retired healthcare professionals, were crucial in alleviating shortages and sustaining staff wellbeing amid high demand and resource constraints [7].

### 4.4. Health Information Systems

The “Tarassud+” platform centralized daily collection of COVID-19 data from government and private sources, enabling real-time monitoring of cases, contacts, and clusters [8,41]. Daily epidemiological reports guided responses, and infection control teams used the “Tarassud+” system to detect healthcare worker infections and outbreaks. Integration challenges, including appointment scheduling, dose tracking, coverage dashboards, price monitoring in the private sector, and traveler vaccination status tracking, arose due to staffing shortages and lack of interoperability between health sectors. Digital contact tracing and geofencing through electronic bracelets were employed to monitor quarantined individuals. However, increasing case numbers, geographic obstacles, and network gaps reduced their effectiveness. In addition, challenges such as high infrastructure costs, sustainability concerns, data security, and community acceptance of AI-tools like chatbots remained [6].

### 4.5. Access to Essential Medicines

Access to essential vaccines was achieved through the establishment of a diverse portfolio via direct purchasing, which reduced the dependency on single suppliers. Every vaccination site had a focal worker who ensured safe handling of the vaccine and adherence to safety and cold-chain protocols. The focal workers were also responsible for keeping accurate records on the number of vaccines/vails used. During vaccine shortages, regional and international partnerships and buffer stock enhanced supply chain resilience [3].

### 4.6. Financing

Vaccination campaigns were fully government-funded and free for citizens and residents, ensuring broad access. However, procurement of vaccines at premium prices strained government budgets, with funding uncertainties arising for booster doses. Sustainable financing options, such as integrating health insurance, may be needed for long-term vaccination programs.

## 5. Strengths, Challenges, and Lessons Learned

Several international studies have applied the WHO Health System Building Blocks framework or related methodological approaches to evaluate COVID-19 vaccine rollouts and health system responses. For instance, WHO guidance documents provide a comprehensive framework for decision-making during global mass vaccination campaigns in COVID-19 pandemic and their implementation [29,49]. Similarly to a recent systematic review, this paper discussed best-practice considerations for mass vaccination campaigns, including coordination, planning, infection prevention and control, vaccination strategies, community engagement, and equitable access [15,22,29].

Despite the methodological limitation of this narrative descriptive review and the potential for reporting bias, the findings and experiences provide valuable insights that can inform and be applied to future public health responses.

The accelerated COVID-19 vaccination campaign in Oman, when assessed through the WHO Health System Building Blocks framework (Table 1), revealed multiple structural and operational weaknesses. Although the MoH maintained strong central coordination, delays in adapting policies to address emerging variants limited the system’s agility. Gaps in public communication, alongside misinformation propagated by false media narratives, underscored the need for active and transparent risk communication. Despite establishing diverse locations for vaccine administration, disparities between rural and urban areas persisted, with remote communities facing slower access. Additional challenges included maintaining the cold chain for mRNA vaccines and combating localized vaccine skepticism. Rapid training and task-shifting enhanced availability of human resources; however, prolonged workloads led to staff burnout, especially in rural regions where trained personnel remained insufficient. The lack of integrated psychological support further exacerbated workforce frustrations. Digital tools such as “Tarassud+” facilitated registration and tracking, but data fragmentation, high operational costs, data security concerns, sustainability beyond emergency phases, and limited interoperability with private healthcare providers reduced the comprehensiveness of reporting [50].

Oman’s diversified vaccine procurement strategy minimized dependence on single suppliers, but global supply chain delays and regional stockouts disrupted their timely distribution. Although universal, government-funded vaccination ensured equitable access, high operational costs placed pressure on budgets, and uncertainty persists regarding sustainable financing for boosters and future campaigns [51]. Similar sustainability concerns about digital health investments and financing exist globally, emphasizing the need for long-term cooperative strategies [50].

## 6. Conclusions

Oman’s accelerated COVID-19 vaccination campaign represents a timely and well-coordinated public health response that substantially mitigated the pandemic’s impact through strong governance, equity-focused strategies, and community trust. However, significant challenges including procurement delays, workforce burnout, data system fragmentation, and budgetary constraints, highlighted areas needing improvement. Despite the descriptive nature of this review, important lessons emerge from this real field experience. Future public health emergencies should prioritize establishing rapid, affordable vaccine procurement mechanisms; integrating comprehensive workforce psychosocial support; investing in interoperable digital health systems for real-time data; expanding inclusive community engagement models; and securing sustainable financing to ensure resilience. These actionable strategies, aligned with the WHO Health System Building Blocks framework, can enhance preparedness, equity, and health security in pandemic responses globally.

## Figures and Tables

**Table 1:** Strengths, challenges, and lessons learned from the accelerated COVID-19 vaccination campaigns in Oman in relation to the WHO Health System Building Blocks framework.

WHO Building Block	Strengths	Challenges	Lessons Learned
**1. *Leadership and Governance***	**Good central coordination** by the MoH ensured a unified strategy. **Inter-ministerial collaboration** facilitated logistics. The healthcare system adapted as Oman shifted resources to manage non-COVID-19 health needs (e.g., chronic diseases) post-vaccination, ensuring holistic care. Global collaboration was ensured through reliance on multiple sources (COVAX and direct purchases from manufacturers), highlighting the importance of international partnerships in vaccine access.	**Delayed policy adjustments** in response to new variants. **False media and public communication gaps** led to misinformation.	**Decentralized decision-making** can improve responsiveness. **Proactive risk communication** is critical to achieving public trust.
**2. *Service Delivery***	**Multi-sectoral vaccination centers** were established, including a field hospital, primary health centers, mobile units, convention centers, schools, and home care, ensuring accessibility to nationals and non-nationals. **Drive-through vaccination sites** enhanced convenience, particularly in urban areas. **The prioritization strategy** effectively targeted high-risk groups first.	**Rural–urban disparities** in access persisted, with remote areas experiencing delays. **Cold-chain logistics** for mRNA vaccines posed difficulties in some regions. **Vaccine hesitancy** in certain communities slowed uptake.	**Mobile vaccination units** are crucial for reaching underserved populations. **Community engagement** is necessary to address hesitancy and misinformation. **Primary healthcare systems** should be strengthened to support future mass campaigns. **Community volunteer work** should be enhanced to combat vaccine hesitancy.
**3. *Health Workforce***	**Rapid training programs** on vaccine administration and safety protocols were implemented for healthcare workers. **Task-shifting** allowed non-physician staff to support vaccination efforts.	**Staff burnout** due to high demand and extended working hours. **Shortages of trained personnel** in rural areas.	**Pre-emptive workforce planning** is essential for surge capacity. **Psychological support** for frontline workers should be integrated into emergency responses.
**4. *Health Information Systems***	**Digital registration platforms** such as “Tarassud+” facilitated appointment scheduling and dose tracking. **Real-time data dashboards** enabled monitoring of vaccination coverage. **Integration of telemedicine within primary care services** helped in reporting side effects and answering public queries.	**Data fragmentation** between different reporting systems across governmental and non-governmental sectors. **Limited interoperability** with private healthcare providers.	**Integrated digital health systems** improve efficiency and transparency. **Regular data audits** ensure accuracy and accountability. **Investment in digital health infrastructure** is important for seamless data integration.
**5. *Access to Essential Medicines (Vaccines and Supplies)***	**A Diversified vaccine portfolio** reduced dependency on a single supplier. **COVAX participation** supplemented national procurement efforts.	**Initial delays in global supply chains** affected rollout timelines. **Stockouts in some regions** due to uneven distribution.	**Local vaccine production or regional partnerships** could enhance supply security. **Buffer stocks** should be maintained for emergency responses.
**6. *Financing***	**Government-funded vaccination** ensured free access for all citizens and residents. **International aid and partnerships** supported vaccine procurement.	**High operational costs** strained healthcare budgets. **Uncertain long-term funding** for booster doses and future campaigns.	**Sustainable financing** mechanisms should be explored. **Emergency health funds** need to be pre-allocated for pandemics. **Dedicated emergency health funds should be established** to achieve rapid responses.

## Data Availability

Not applicable.

## Abbreviations

The following abbreviations are used in this manuscript:

WHO	World Health Organization
EVM	Effective Vaccine Management
MoH	Ministry of Health
PHC	Primary Healthcare
HCWs	Healthcare Workers
